# Insights into high-risk multiple myeloma from an analysis of the role of PHF19 in cancer

**DOI:** 10.1186/s13046-021-02185-1

**Published:** 2021-12-02

**Authors:** Hussein Ghamlouch, Eileen M. Boyle, Patrick Blaney, Yubao Wang, Jinyoung Choi, Louis Williams, Michael Bauer, Daniel Auclair, Benedetto Bruno, Brian A. Walker, Faith E. Davies, Gareth J. Morgan

**Affiliations:** 1 Myeloma Research Program, NYU Langone Medical Center, Perlmutter Cancer Center, 522 1st Avenue, Manhattan, New York City, NY 10016 USA; 2 Applied Bioinformatics Laboratories (ABL), NYU Langone Medical Center, New York, NY USA; 3 Department of Biomedical Informatics, University of Arkansas for Medical Sciences, Little Rock, AR USA; 4 The Multiple Myeloma Research Foundation (MMRF), Norwalk, CT USA; 5 Division of Hematology Oncology, Indiana University, Indianapolis, IN USA

**Keywords:** Multiple Myeloma, PHF19, Polycomb Repressive Complex 2, PRC2, EZH2, Epigenetic, Cancer progression

## Abstract

Despite  improvements in outcome, 15-25% of newly diagnosed multiple myeloma (MM) patients have treatment resistant high-risk (HR) disease with a poor survival. The lack of a genetic basis for HR has focused attention on the role played by epigenetic changes. Aberrant expression and somatic mutations affecting genes involved in the regulation of tri-methylation of the lysine (K) 27 on histone 3 H3 (H3K27me3) are common in cancer. H3K27me3 is catalyzed by EZH2, the catalytic subunit of the Polycomb Repressive Complex 2 (PRC2). The deregulation of H3K27me3 has been shown to be involved in oncogenic transformation and tumor progression in a variety of hematological malignancies including MM. Recently we have shown that aberrant overexpression of the PRC2 subunit PHD Finger Protein 19 (PHF19) is the most significant overall contributor to HR status further focusing attention on the role played by epigenetic change in MM. By modulating both the PRC2/EZH2 catalytic activity and recruitment, PHF19 regulates the expression of key genes involved in cell growth and differentiation. Here we review the expression, regulation and function of PHF19 both in normal and the pathological contexts of solid cancers and MM. We present evidence that strongly implicates PHF19 in the regulation of genes important in cell cycle and the genetic stability of MM cells making it highly relevant to HR MM behavior. A detailed understanding of the normal and pathological functions of PHF19 will allow us to design therapeutic strategies able to target aggressive subsets of MM.

**Supplementary Information:**

The online version contains supplementary material available at 10.1186/s13046-021-02185-1.

## Background

Multiple myeloma (MM) is a hematopoietic malignancy of terminally differentiated plasma cells (PC) [1]. Multiple myeloma comprises 1.5% of all malignant diseases and accounts for 10% of all hematologic malignancies [2]. Despite the advent of novel therapies, MM remains difficult to treat and contributes to 20% of the deaths from hematologic malignancy. Recurrent relapses and increasingly aggressive drug resistant disease that grows outside the marrow cavity are typical features of MM progression [1]. The clinical stage of MM is preceded by a premalignant expansion of clonal plasma cells, recognized clinically as monoclonal gammopathy of undetermined significance (MGUS) and/or smoldering MM (SMM) and that have been shown to transform into MM, plasma cell leukemia (PCL) or extramedullary disease over time [3]. On average, 1% of cases of MGUS and 5-7% of SMM transform to MM annually; rates that are greater for higher-risk subgroups. At presentation, 15-25% of MM cases have high-risk (HR) disease that is associated with early relapse and high mortality rates. The percentage of patients with HR features increases at each relapse constituting a major therapeutic challenge. Although genetic features associated with HR have been identified none explain the majority of disease and epigenetic modifications including DNA methylation, chromatin accessibility, and histone modifications seem to play an important role and may also be therapeutically tractable [4–6].

Epigenetic variables include post transcriptional modification of histones that play an important role in the regulation of genes expression by recruiting transcription factors (TFs) and affecting chromosome structure and function [7, 8]. These modifications include phosphorylation on serine or threonine residues, methylation on lysine or arginine together with other modifications such as acetylation, ubiquitylation or sumoylation. Histone methylation is mediated by specific methyltransferases that add methyl groups to lysine (mono-, di-, and trimethyl), glutamate, or arginine residue. The tri-methylation of the lysine (K) 27 on histone 3 H3 (H3K27) is a critical determinant of chromatin accessibility required for gene expression and is regulated by the proteins of the polycomb group (PcG) [7, 8]. These polycomb proteins are an evolutionarily conserved group that regulate gene expression through histone modification brought about by their interaction to form chromatin-associated repressive complexes: Polycomb-repressive complex 1 (PRC1) is responsible for the ubiquitylation of the lysine 119 on histone 2A H2AK119ub1, and PRC2, catalyzes the methylation of H3K27.

Aberrant expression and somatic mutations affecting genes involved in the regulation of H3K27me3 deposition/removal are common in cancer. The deregulation of H3K27me3 has been shown to be involved in oncogenic transformation and tumor progression in a variety of hematological malignancies including MM [9–11]. In both MGUS and MM, PRC2 target genes, identified by their H3K27me3 marks, have been shown to decrease their level of expression as disease progresses [5, 6]. PRC2-mediated gene silencing as a mechanism of gene repression during MM progression has also been highlighted by genome-wide profiling of H3K27me3 marks combined with RNA-Seq [12]. This analysis identified increased silencing of H3K27me3 targets in MM patients at advanced stages of the disease and the expression of H3K27me3-marked genes correlated with poor patient survival [12]. Pharmacological inhibition of Enhancer of Zeste Homolog (EZH) 2 has anti-myeloma effects in both MM cell lines and CD138+ MM patient cells [1, 12]. These studies suggest that the mechanisms that control PRC2/EZH2 activity might serve as a novel therapeutic target for MM.

Polycomb repressive complex 2 (PRC2) is a multi-subunit epigenetic protein complex that regulates gene expression by catalyzing mono-, di- and tri-methylation of histone H3 on lysine 27 (H3K27me1, H3K27me2, and H3K27me3, respectively) and plays a role in safeguarding cellular identity by ensuring gene silencing appropriate for the cellular function at that stage of development [7, 8, 13]. PRC2 is comprised of the core subunits EZH1 or EZH2, Embryonic ectoderm development (EED) and Suppressor of zeste 12 (SUZ12), and RBBP4/7 [7, 8]. EZH1 and EZH2 are the catalytic subunits of PRC2 and are mutually exclusive within the complex retaining distinct enzymatic properties *in-vitro*, with EZH2 having higher methyltransferase efficiency under the same reaction conditions [14]. PRC2 activity can be modulated by different accessory subunits including PHF1, MTF2, PHF19, PALI1, EPOP, JARID2 and AEBP2 [7, 8]. Some of these accessory subunits display cell type–specific expression patterns, and some exist within a subset of the PRC2 complex. They are not essential for the basal activity of PRC2 but may direct PRC2 recruitment or modulate PRC2 activity under specific conditions [7, 8]. Recently, we have shown that aberrant overexpression of the PRC2 subunit *PHF19* is the most significant overall contributor to HR status focusing attention on the role played by epigenetic change and PRC2 behavior in aggressive clinical states of MM [15, 16]. In this review, we discuss the current knowledge on the regulation of PRC2/EZH2 activity by PHF19, its biological impact in MM and the potential for anti-PHF19 targeted therapy.

## Structure and function of PHF19 protein

The core of the PRC2 complex can interact with three paralogues of the Drosophila polycomb-like gene, Plant Homeodomain (PHD) Finger Protein (PHF)1/PCL1, MTF2/PCL2 and PHF19/PCL3 that modulate PRC2 enzymatic activities and recruitment to target genes important for development and differentiation [17–21].

**Fig. 1:**
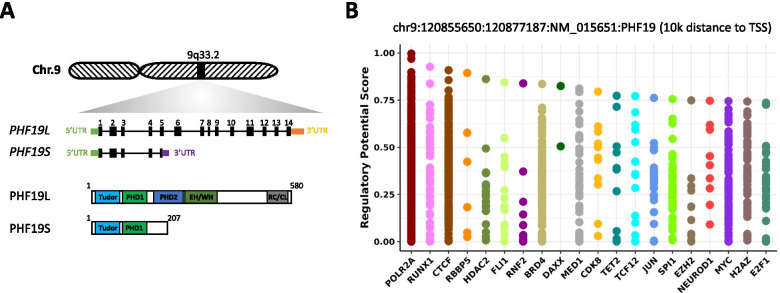
Human PHF19 isoforms and Top 20 TFs and chromatin regulators that regulate *PHF19* expression in cancer. **A** Schematic representation of *PHF19* locus, gene and isoforms. **B** The Cistrome DB Toolkit (http://dbtoolkit.cistrome.org/) was used to identify what TF likely regulates *PHF19* expression spanning a region of ∼10 kb upstream the transcription start site (TSS). The Y axis represents the regulatory potential score which were calculated by Cistrome DB Toolkit [23, 24]. The x-axis represents the different TFs

The human *PHF19* locus is located in chromosome 9q33.2 and was initially identified near a retroviral integration site in an immortalized human fibroblast cell line [22]. It has been suggested that the gene is expressed as multiple, alternatively spliced mRNAs with at least three different 5′ ends and that two types of mRNA are derived from the *PHF19* gene encoding short (PHF19S, 207 aa) and long (PHF19L, 580 aa) isoforms that share 155 amino acids at their N-termini [22] (Fig. 1A). The *Ensembl* database reports 14 transcripts, three retained-intron transcripts, three processed transcripts and eight protein coding transcripts (Supplementary Table 1). The NCBI database reports 18 predicted transcripts produced either by NCBI’s genome annotation pipeline or copied from computationally annotated submissions to the International Nucleotide Sequence Database Collaboration (INSDC). However, subsequently curated RefSeq NCBI records report five protein coding transcripts (Supplementary Table 1). Human *PHF19S* mRNA is one out of 23 human gene mRNA that contain a ‘readthrough’ stop codon (these mRNAs have a UGA stop codon immediately followed by CUAG) [25]. This means that the ribosomes translating the *PHF19S* mRNA decode its UGA stop codon as a sense codon, thus extending PHF19S at its C terminus by an additional 156 amino acids to generate a protein of approximately 40 kDa. The readthrough efficiency for *PHF19S* mRNA was estimated to be around 2% in HEK293 cell line [25] but no data are available in cancers. The Human Protein Atlas database reports eight PHF19 isoforms (Supplementary Table 1). Cellular localization analysis of PHF19L and PHF19S isoforms showed that the long isoform is exclusively nuclear and that the short isoform is localized in both cytosol and the nucleus [22, 26]. Two putative nuclear localization signals are predicted to be located at amino acids 387–397 and 473–489 of the long isoform [22, 26].

PHF19 contains an amino terminal Tudor domain and two PHD domains, one EH/WH (Extended Homology/Winged-Helix) domain and a C-terminal RC/CL (Reversed Chromo/Chromo-Like) domain [20, 21]. PHF19S lacks one of the second PHD-domains and the EH/WH domain and contains a specific C-terminal region (Fig. 1A). Of note, the murine *Phf19* locus encodes only a long isoform (Phf19L, 578aa) with 90% homology with the human PHF19L protein sequence and with conserved Tudor, PHDs, EH/WH and RC/CL domains. In human embryonic kidney cells (HEK293T), both PHF19S and PHF19L have been shown to associate with the PRC2 components EZH2 and EED but not within the same complex [26]. The EH/WH domain of PHF19 interacts with unmethylated CpG DNA islands at promotors, enhancing the chromatin association of PRC2 [27]. The RC/CL domain of PHF19L is necessary for the interaction with SUZ12 and to stabilize the dimeric state of PRC2 promoting chromatin binding, possibly through an avidity effect [28]. The PHF19 Tudor domain has been shown to bind to H3K36me3 suggesting that it targets the PRC2 complex to the chromatin of actively transcribed genes [20, 29, 30]. H3K36me3 marks have been shown to inhibit the ability of PRC2 to methylate H3K27 [31]. PHF19 facilitates the removal of H3K36me3 through the recruitment of an H3K36me3 demethylase, NO66 or KDM2B [20, 29]. PHF19 has been shown to physically interact with NO66 [29] and to co-localize on the chromatin with the H3K36me2 demethylase KDM2B [20]. However, ChIP-Seq profiling data obtained from mouse embryonic stem cells (mESCs) showed a moderate genome-wide association between Phf19 and H3K36me3 [21, 30]. PHF19 may also be able to bind to H3K27me3, although with a lower affinity than it binds to H3K36me3 [29, 32]. A subset of PRC2 target genes with bivalent H3K36me3 and H3K27me3 colocalized with Phf19 in mESCs and F9 embryonic carcinoma cells [30]. Interestingly, Phf19 has been shown to localize at bivalent promotor marked by both H3K4me3 and H3K27me3 in mESCs [21, 29] suggesting that it may target PRC2 to genes involved in cell differentiation or cell cycle.

Phf19 function has been characterized mainly in mESCs were it is dispensable for their self-renewal, it potentiates their differentiation and is required for the silencing of embryonic stem cell genes. In mESCs, Phf19 has been shown to localize with about 60-80% of SUZ12 binding sites [20, 21, 29, 30]. Phf19 colocalizes with another PCL protein MTF2 at a subset of PRC2 target genes [27]. shRNA-mediated *Phf19* knockdown (KD) in mESCs leads to de-repression of a subset of PRC2 target genes involved in self-renewal, pluripotency and differentiation [21, 29]. These functions also correlate with a decrease in H3K27me3 at a subset of these genes [20, 21, 29, 30]. Mutation of conserved residues within the Phf19 Tudor domain, a domain implicated in recognizing H3K36me3, led to similar results [21, 30]. Overexpressing Phf19 in mESCs leads to enhanced global H3K27me3 levels [21]. Overall, these data suggest that in mESCs, Phf19 plays a role in PRC2 stabilization and in the spreading of PRC2/H3K27me3 to adjacent H3K36me3/2-positive nucleosomes after initial recruitment of PRC2 to the promotor by other PRC2 cofactors including Eed, Jarid2 or non-coding RNA [20, 21, 29, 30]. However, one should note that in mESCs, Phf19 is expressed approximately tenfold lower relative to Mtf2 and stoichiometry analysis of the different PRC2 complexes shows the number of PRC2 complexes containing Phf19 is minimal compared with Mtf2-containing complexes [33]. Furthermore, unlike in human cells, mouse cells lack the PHF19 short isoform that contains both the Tudor domain and the first PHD domain and is present in both cytoplasm and the nucleus of cells. These features suggest that the role of the PHF19L in recruiting PRC2 to target sites may be conserved between mice and human, however, the presence of the PHF19 short isoform may interfere or cooperate with the function of the PHF19L isoform leading to different roles and molecular mechanisms.

In normal tissue, analysis of *PHF19* mRNAs by Northern blot showed that it is most abundant expression is in the thymus and heart, whereas they were barely detected in the lung and kidney [22]. RNA-Seq data shows that *PHF19* expression is high in the artery, bone marrow and lymphoid tissues and the lowest in the pancreas and the liver (The Human Protein Atlas, https://www.proteinatlas.org/ENSG00000119403-PHF19/tissue). The ratio of *PHF19S* to *PHF19L* mRNAs varies among different tissues [22]. Placenta, skeletal muscle, and kidney express predominantly *PHF19L* mRNA, whereas liver and peripheral blood leukocytes contain more *PHF19S* mRNA [22]. In the mouse, *Phf19* is expressed at low levels in all hematopoietic cells; however, differences in expression levels between stem, progenitor and mature progenitors are observed. *Phf19* expression is modulated during activation and differentiation of mature blood cells such as B-cells and T-cells (see below). In normal mouse T-cells, the AKT pathway was shown to regulate the expression of *Phf19* [34]. Increased Akt phosphorylation and activity through the downregulation of the Akt phosphatase Ship1 by miR-155 induces *Phf19* transcription [34]. Further, the pharmacological inhibition of Akt in T-cells led to decreased expression of *Phf19* [34].

## Alterations of *PHF19* expression in cancer

*PHF19* is overexpressed in a number of human cancers including prostate cancer [35], glioblastoma, [36, 37], hepatocellular carcinoma [30, 38–40], ovarian cancer [41, 42], melanoma [22, 43], gastric cancer [44–46]. EWS/ETS-driven Ewing sarcoma [47] and colorectal cancer [48]. Using overexpression and KD of *PHF19* several studies have shown that PHF19 is implicated in aggressive clinical behavior associated with increased proliferation, migration and invasion. The findings of these studies are summarized in Table 1. However, to date a global comprehensive analysis aimed at identifying the genetic targets and pathways controlled by PHF19 in cancer has not been reported (except one study in a prostate cancer cell line [35]).

**Table 1:** Alterations of PHF19 expression in cancer

Cancer type	Clinical impact	Experimental method	Effect of PHF19 overexpression on the tumor biology	Reference
**Glioblastoma**	*PHF19* expression is higher in later stages of disease and higher expression correlates with poor survival.	shRNA knockdown of PHF19L and S and rescue experiment in cell lines.	PHF19 promotes proliferation, migration, and resistance to doxorubicin through the suppression of the expression of the β-catenin ubiquitination ligase SIAH1 and the regulation of the expression of genes involved in cell cycle (CDK4, CDK6, c-MYC and Cyclin D1) and migration.	[35, 36]
**Hepatocellular carcinoma**	High expression of *PHF19* correlates with poor survival.	MYC-driven hepatocellular carcinoma mouse model. shRNA knockdownand PHF19 overexpression in cell lines.	Depletion of *Phf19* in a MYC-driven hepatocellular carcinoma mouse model significantly decreased tumor burden and improved mouse survival. Overexpression of PHF19S in a KRasG12D/P53KO-driven hepatocellular carcinoma mouse model significantly promoted tumorigenesis and metastasis. PHF19 regulated the β-catenin pathway, through impairing the ubiquitination of β-catenin.	[30, 37–39]
**Ovarian cancer**	N.A.	Xenograft assay. shRNA knockdown of PHF19L in cell lines.	shRNA mediated PHF19 KD lead to diminished cell proliferation, invasion, migration, and stemness of cell lines in vitro. In vivo xenograft assay of an ovarian cancer cell line showed that PHF19L KD significantly suppressed tumor growth.	[40, 41]
**Melanoma**	N.A.	siRNA knockdown of PHF19L and S in cell lines.	PHF19 promotes cell invasion and cell proliferation through upregulating the expression of Cyclin D1 and Cyclin A. contrary to what was observed in ovarian cancer cell line, PHF19 KD did not affect the expression of stemness genes (NANOG and OCT4).	[22, 42]
**Gastric cancer**	*PHF19* is upregulated in gastric cancer tissues compared with adjacent normal stomach tissues and correlates with tumor cell differentiation state and poor outcome.	Xenograft assay. shRNA knockdown of PHF19L in cell lines.	PHF19L promotes cell growth, colony formation and migration in vitro and tumor growth in vivo. PHF19L promotes AKT and ERK activity through regulating phosphorylation of both kinases and its overexpression increases the expression of Cyclin D1 and Cyclin E.	[43–45]
**EWS/ETS-driven Ewing sarcoma**	High levels of *PHF19* expression significantly correlates with overall survival rates. The PHF19 levels in high-risk patients are significantly upregulated compared to the low-risk patients.	CRISPR-Cas9–mediated knockout of PHF19L in cell lines.	PHF19 was identified as a target for EWS/FLI1 or EWS/ERG fusions and BRD4. PHF19-KO cells result in a significant reduction in proliferation, colony forming ability, and invasive potential and showed higher sensitivity to BRD4 inhibitor JQ1.	[46]
**Colorectal cancer**	*PHF19* is significantly overexpressed in tumors compared with normal tissues and it is high expression associate with poor overall survival, tumor progression and metastasis.	Overexpression of PHF19 in cell lines.	PHF19 overexpression mediate cell cycle progression. PHF19 overexpression led to increased protein levels of Cyclin D1, CDK4, and CDK6 and to increased percentage of cells in the S phase of the cell cycle.	[47]
**Prostate cancer**	see text.	see text.	see text.	see text.

*N.A.* Not available

In prostate cancer, two studies were published showing controversial data regarding the cellular localization and the role of PHF19S in cell growth and migration of the same cell lines (DU145 and PC3) [35, 49]. The mRNA level of PHF19S but not PHF19L was shown to be higher in cancer tissues compared with adjacent normal tissues [49]. In two androgen-independent prostate cancer cell lines (DU145 and PC3) compared to a non-tumorigenic prostate epithelial cell line (RWPE-1) both PHF19L and PHF19S were seen to be overexpressed by western blot [35, 49]. The analysis of cellular localization by cell fractionation showed that, PHF19S is only cytoplasmic in one study [35], however; in the other study [49] PHF19S was shown to be present in both cytoplasm and nucleus. There was further contradictory data using ShRNA against PHF19S, with one study [35], showing that PHF19S KD had no impact on the proliferation or migration of DU145 and PC3 cell lines, whereas the other [49] showed that PHF19S KD significantly impaired proliferation and migration in both cell lines. This impact on proliferation of PHF19S was shown to be dependent on its PHD1 domain but not on its Tudor domain or its specific C-terminal domain [49]. Similar to hepatocellular carcinoma, PHF19S seems to regulate IL6 expression in prostate cancer but is not involved in the regulation of β-catenin degradation [49]. The study by Jain et al. [35] then focused on PHF19L and showed that it is KD in DU145 and PC3 cell lines performed impaired cell proliferation. Whereas the idea that PHF19 has a general role as a transcriptional repressor as part of the PRC2 complex is legitimate, RNA-seq analysis of PHF19L KD vs control (p value ≤ 0.05; FC ≥ 1.4) in DU145 cell line showed 1499 differentially expressed genes, among them 652 upregulated genes (40%) and 847 downregulated genes (60%) suggesting rather that PHF19L is a transcriptional activator in this cell line. Gene Ontology analysis showed that upregulated genes are involved in regulation of serine/threonine kinase activity, response to wounding, response to oxygen levels and that genes downregulated are involved in response to type 1 interferon, embryonic organ development and regulation of cell projection organization [35]. Similar to other cancer (e.g. glioblastoma and gastric cancer) PHF19L seems to be involved in the expression regulation of genes involved in cell cycle [35]. PHF19L KD showed downregulation of the mRNA of Cyclin A2, Cyclin B2, CDK4 and E2F1, however, the mRNA expression of p21/CDKN1A was increased [35]. Rescue experiments by stably overexpressing PHF19L in KD cells restored the expression of these genes to the WT level suggesting that PHF19L specifically regulate the expression of these genes [35]. ChIP-seq analysis showed that depletion of PHF19L lead to a genome-wide gain in PRC2 occupancy and H3K27me3 deposition which may suggest that PHF19L have an inhibitory impact on PRC2 activity, however the authors suggest that MTF2 compensates for the loss of PHF19 through increasing chromatin recruitment of PRC2 [35].

In chronic myeloid leukemia (CML), high expression of PHF19 correlates with adverse clinical outcomes [50]. PHF19L KD in three human myeloid leukemia cell lines (K562, HL60 and NB4) showed a significant impact on cell proliferation but not apoptosis [50]. Cell cycle analysis of PHF19L KD cells showed a significant blockage in phases G1 and G2-M and reduction of S-phase of the cell cycle [50]. Transcriptomic analysis following PHF19L depletion showed downregulation of the MYC network, regulation of G1/S transition of mitotic cell cycle and G2/M checkpoint genes sets [50]. Once more, Cyclin D1 and p21/*CDKN1A* were shown to be downregulated and upregulated, respectively, following *PHF19* depletion [50]. In this regard, PHF19 was shown to localize at the transcription start site of the *CDKN1A* gene [50]. ChIP-seq experiment shows that 90% of PHF19 target genes were shared with PRC2-targets (shared among EZH2, SUZ12 and H3K27me3), and about 10% out of PRC2-target genes [50]. Despite this colocalization with PRC2, PHF19L depletion led to a significant number of downregulated genes, suggesting an ambiguous role for PHF19 in the regulation of gene expression. It has been shown that MTF2 could compensate for PHF19 loss at some loci, but the authors clearly state that the general de-repression of PHF19 target genes upon depletion of PHF19 is incompatible with MTF2 compensation and that at some genes the lack of PHF19 directly leads to the upregulation of gene expression [50].

Thus, despite some conflicting results, PHF19 seems to play a role in the proliferation of cancer cells by regulating the expression of cell cycle-associated factors. *PHF19* KD experiments in several cancer cellular models shows the downregulation and de-repression of the expression of a significant number of PRC2-target genes, however, a number of genes have no detectable change in expression. These data suggest that PHF19 might play a mixed role in regulating PRC2-target genes expression and that other PCL proteins may compensate for PHF19 loss at some PRC2-target genes. For example, PHF19 promotes the expression of cell cycle activator including cyclins (e.g. Cyclin D1), and cyclin-dependent kinases (CDKs) and suppresses the expression of cyclin-dependent kinase inhibitors (e.g. CDKN1A). These data indicate that PHF19 either plays a role in the context-dependent regulation of PRC2 activity or alternatively that it may have activity independent of PRC2.

## The regulation of *PHF19* expression in cancer cells

Currently, the transcriptional, post-transcriptional and post-translational regulation of PHF19 in normal and cancer cells is underexplored; however, some insights are available from cancer cells. In melanoma cell lines, pharmacological AKT inhibition leads to decreased expression of PHF19 protein [43]. Using immunofluorescence and ChIP-qPCR it has been shown that pAKT is present in the nucleus of the human melanoma cell line HT144 and that it localizes to the promotor of *PHF19* [43]. EWS/FLI1 fusion in Ewing sarcoma was shown to regulate *PHF19* expression by collaborating with BET proteins and the binding of a distal regulatory element (enhancer) of *PHF19* [47]. EWS/FLI1 knockdown or BET inhibitor treatment resulted in downregulation of *PHF19* expression [47]. In a pancreatic cancer cell line, the H3K9 methyltransferase G9a was shown to bind the promotor of *PHF19* and to promote it is expression; however, the mechanism is not clear [51]. G9a knockdown or pharmacological inhibition resulted in downregulation of *PHF19* expression [51]. Using Toolkit for Cistrome Data Browser [23, 24] that calculates a regulatory potential (RP) score for a given gene using TF ChIP-seq samples from cancer and normal cells, we show that the top 20 TFs and chromatin regulators that regulate *PHF19* expression in a region up to 10 kilobase from the TSS include RUNX1, FLI1, SPI1, MYC, E2F1, CTCF, HDAC2, TET2 and EZH2 (Fig. 1B). At a post-transcriptional level, *PHF19* expression was shown to be regulated by several MicroRNA (miRNA) including miR-211 (in ovarian carcinoma [42]), miR-497 and miR-195-5p (in hepatocellular carcinoma [38, 39]), miR-15a (in gastric cancer [46] and MM [52, 53]).

## PHF19 in hematopoiesis

### PHF19 balance quiescence and differentiation of hematopoietic stem cells (HSCs)

**Fig. 2:**
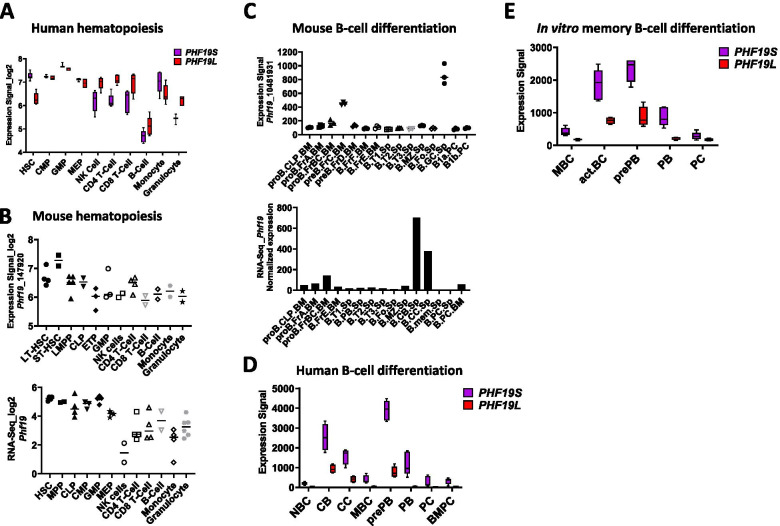
*PHF19* expression in normal human and mouse hematopoiesis in normal human and mouse B-cell differentiation. *PHF19* expression in human (**A**) and mouse (**B**) expression profiles in normal hematopoiesis as reported in the Blood Spot database [54] (http://servers.binf.ku.dk/bloodspot/, accessed on 27 July 2021). **A** the Normal human hematopoiesis (HemaExplorer) dataset was used. **B** the Mouse normal hematopoietic system (upper) and Mouse Normal RNA-Seq (Lower) datasets were used. LT-HSC: Long Term Hematopoietic Stem Cell, ST-HSC: Short term Hematopoietic stem cell, HSC: Hematopoietic Stem Cell, LMPP: Lymphoid-primed multipotential progenitors, MPP: Multipotent Progenitor, CLP: Common Lymphoid Progenitor, CMP: Common Myeloid Progenitor, GMP: Granulocyte Monocyte Progenitor. *PHF19* expression in mouse (**C**) and human (**D and E**) expression profiles in normal human and mouse B-cell differentiation. **C** *Phf19* expression as reported in the Blood Spot database [54] (http://servers.binf.ku.dk/bloodspot/, accessed on 27 July 2021) using the Mouse immgen B cells dataset (upper) and as reported in Immgen dataset (http://rstats.immgen.org/Skyline/skyline.html, accessed on 27 July 2021) using RNA-seq Gene Skyline tool (Lower). **D and E** *PHF19* expression as reported in the GenomicScape database [55] (http://genomicscape.com/microarray/expression.php, accessed on 27 July 2021) using the Human B cells to plasma cells GCRMA dataset (**D**) and Human B cells to plasma cells (in-vitro) dataset (**E**). NBC: Naive B cells (*n*=5), CB: Centroblasts (*n*=4), CC: Centrocytes (*n*=4), MBC: Memory B cells (*n*=5), prePB: preplasmablasts (*n*=5), PB: Plasmablasts (*n*=5), PC: Early plasma cells (*n*=5), BMPC: Bone marrow plasma cells (*n*=5), act.BC: Activated B-cells (*n*=5)

In human hematopoiesis, *PHF19S* expression is higher in stem/progenitor cells and relatively decreases during differentiation (except in monocytes); however, *PHF19L* expression seems to maintain similar levels between progenitors and mature differentiated cells (except in B-cells) (Fig. 2A). Among human mature cells, *PHF19S* expression is highest in human CD14+ monocytes and *PHF19L* is the highest in NK and T cells; however, peripheral blood B-cells express the lowest levels of both isoforms (Fig. 2A). In comparison with *PHF19S* level, *PHF19L* level is high in peripheral blood T-cells, B-cells, NK cells and granulocytes but not monocytes (Fig. 2A). In mouse hematopoiesis, *Phf19* expression is relatively elevated in undifferentiated progenitors and progressively decreases during differentiation (Fig. 2B). Constitutive *Phf19*-KO mice have an increase in HSCs embryonic fetal liver and a decrease in adult bone morrow HSC that exhibit a quiescent phenotype and a differentiation defect [56]. Young constitutive *Phf19*-deficient mice exhibit normal T cell development and homeostasis [34], however, aged *Phf19*-deficient mice (>60 weeks) have a high penetrance of splenomegaly [56]. Phf19 is suggested to play a role in long-term hematopoietic reconstitution as serial non-competitive bone-morrow transplantation experiments of *Phf19*-KO cells show reduced repopulation capacity and diminished ability to produce new colonies over passages [56]. Transplanted mice show a myeloid lineage differentiation bias and develop splenomegaly with increased accumulation of myeloid cells and higher rate of proliferation suggesting that *Phf19* is necessary to maintain a correct hematopoietic balance and that its depletion primes HSCs for malignant progression [56]. RNA sequencing analysis on *Phf19*-deficient HSCs showed enrichment for genes of HSC identity and downregulation of the Myc network, which is required for HSC differentiation [56]. ChIP-seq and ATAC-seq experiments in HSCs showed a surprising global increase in H3K27me3 levels and chromatin compaction at genes characteristic of differentiated lineages and at TFs genes implicated in HSC differentiation into erythroid, granulocyte, lymphoid or megakaryocyte [56]. This increase in H3K27me3 levels was associated with a downregulation of the expression of TF involved in HSC differentiation [56]. These data show that Phf19 promotes the differentiation of HSC and decreases their identity and quiescence by the regulation of expression of lineage specific differentiation genes and TFs, similar to its role in other types of stem cell such as mESC.

Although it is difficult to compare data on hematopoiesis between different transgenic mice models of PRC2-component loss (i.e. the developmental stage at which the KO is induced using Cre), the impact of *Phf19*-loss on HSCs shows some differences by comparison with published results for other PRC2 subunits [9, 57–60]. For example, Ezh2 is dispensable for maintenance of long-term LT-HSCs and Ezh2-deficient HSCs largely preserve normal differentiation and function and do not show de-repression of cell cycle and differentiation inhibitor genes [9]. Nevertheless, combined deletion of Ezh1 and Ezh2 abolished the repopulating capacity of HSC [9, 57]. However, the depletion of either Suz12 or Eed results in HSC exhaustion at the fetal or adult stage showing that Suz12 is essential for T and B cell maturation but is dispensable for correct myelopoiesis [9, 58, 59]. Depletion of the PRC2 accessory subunit, Jarid2, enhances the competitive transplantation capacity of both fetal and adult mouse HSPCs [9, 60]. Mtf2 was shown to be important for erythropoiesis and proper PRC2 recruitment to erythrocyte maturation regulator genes [61]. In the human CML cell line model, K562, that could be differentiated toward erythroid or megakaryocyte cell fate, *PHF19* depletion enhanced erythroid differentiation while impeding megakaryocyte cell fate induction [50] suggesting that *PHF19* might be important for lineage differentiation from bipotent megakaryocyte-erythrocyte progenitors.

Overall, these data indicate that PCR2 accessory subunits including PHF19, may have specific functional roles in recruiting the PRC2 complex to specific genomic targets involved in the long-term maintenance of hematopoiesis, differentiation and specification of blood-lineage. Despite few overlapping phenotypes with other PCL factors, these data indicate that in hematopoiesis Phf19 has a specific role in regulating HSC quiescence and differentiation to pan-blood lineage.

### PHF19 regulates CD8+ T-cell effector function

The role of Phf19 was investigated in mouse CD8+ T-cells differentiation and function [34]. Phf19 was shown to orchestrate a transcriptional program able to restrain T cell senescence and to sustain CD8+ T-cell antitumor and immune responses [34]. Phf19 prevents terminal differentiation of T-cells through epigenetic silencing of the pro-effector and pro-exhaustion TFs including Eomes, Id2, Maf and Prdm1 [34]. In an infection model with recombinant vaccinia virus expressing the human glycoprotein 100 (gp100), *Phf19* expression in CD8+ T-cells, similar to that of *Ezh2*, was strongly induced at the early stages (first 2 days) of the acute immune response, sharply downregulated at peak effector response and maintained at low levels throughout transition to memory phase [34]. In a mouse model of antitumor function, Phf19 overexpressing T-cells displayed limited senescence and sustained cytokine production and anti-tumor activity resulting in increased mice survival compared to controls [34]. This phenotype was dependent on the ability of Phf19 to modulate PRC2/EZH2 activity as Phf19 with mutated Tudor domain or it is overexpression in Ezh2-deficient T-cells abolished the phenotype [34]. These data have implication for cancer immunotherapy through epigenetic reprogramming of CD8+ T cell fate where Phf19 expression promotes effector function of these cells. These data suggest that in mature specialized blood cells (ex. CD8+ T-cells), Phf19 regulate PRC2 activity at genes involved in specialized function of these cells.

## PHF19 in B-cell and plasma cells differentiation

In contrast to EZH2, the role of PHF19 in B-cell and plasma cell development and differentiation is not explored. Gene expression data shows that *PHF19* is expressed at low levels in quiescent B-cells; however, its expression is upregulated in activated, proliferating, and fate-committed cells. In mouse B-cells, *Phf19* expression is the highest in cycling pre-B (Fraction. Cprime, Fr.C) and germinal center (GC) B-cells (Fig. 2C). In human mature B-cells, *PHF19* expression is highest in GC B-cells (centroblasts) and preplasmablasts (Fig. 2D). *PHF19S* expression is significantly higher than *PHF19L* expression in all B-cell and PC subsets (Fig. 2D).

### PRC2/EZH2 in germinal center and PC differentiation

The germinal center reaction plays a critical role in myeloma pathogenesis. The PRC2 complex is an important mediator of the germinal center reaction and normal plasma cell differentiation; therefore, as a PRC2 activity regulator, PHF19 potentially plays an important role in these processes. Germinal centers are micro-structures that develop in secondary lymphoid organs as a result of B-cell activation by a specific antigen and the interaction with antigen-specific T-cells. This interaction allows the amplification and proliferation of B-cells, ensures high affinity antibody creation and the generation of memory B-cells or long-lived antibody secreting plasma cells. In mice, Ezh2, is required for GC formation *in-vivo* and promotes the proliferation of GC B-cells *ex-vivo* and its depletion leads to impaired PC production [62–64]. Ezh2 physically interacts with Bcl6 and represses PC differentiation via the suppression of Blimp1 and Irf4 transcription [65]. Ezh2 promotes the proliferation of GC B-cells through the suppression of the cell cycle inhibitors, Cdkn1a, Cdkn2a and Cdkn1b [66]. However, how Ezh2 is recruited to its target genes in GC B-cells and whether Phf19 play a role in this is unknown. EZH2 is actively involved in the de-novo establishment of bivalency at monovalent H3K4me3 promoters of GC exit genes [65]. This bivalent state at the gene promotors leads to a transient repressive state from which they can either become activated or stably repressed, depending on their differentiation commitment [65]. *EZH2* gain-of-function mutations are among the most common genetic alterations identified in GC-derived B-cell lymphoma [62, 65]. Mutated *EZH2* in B-cell lymphoma enables persistent epigenetic silencing of genes involved in PC differentiation and negative regulation of cell cycle [62, 66]. In MM, EZH2 is overexpressed and associated with adverse prognosis but is generally not mutated [4, 5].

During terminal differentiation into mature non-proliferating PCs, the GC B-cells or activated memory B-cells differentiate into plasmablasts that retain a proliferative capability in the context of a PC phenotype. A precursor population for plasmablast called a pre-plasmablast was first identified during the generation of plasma cells from human memory B cells *in vitro* [67, 68]. An *in-vivo* counterpart of the *in vitro* generated pre-plasmablasts was identified in human lymph nodes and tonsils [68, 69]. Interestingly, pre-plasmablasts co-express B and plasma cell TFs at low levels (BCL6, PAX5, IRF4, Blimp-1, XBP1) [68]. In this cell type, EZH2 seems to directly regulate the expression of genes involved in cell cycle (*E2F7*, *CCNA2*, *E2F1*, or *AURKB*) and DNA replication (including *CDT1*, *POLD1*, *CDC45*, *MCM2*, and *MCM5*) as these gene loci are enriched for EZH2, however, genes involved in memory B-cell phenotype maintenance seems to be an indirect target for EZH2 [70]. Interestingly, in this study EZH2 was found to be recruited to H3K27me3-free promoters of transcriptionally active genes known to regulate cell proliferation including *AURKA*, *MCM5*, *CCND2* and *RAD51AP1* [70]. The absence of H3K27me3 at the promotor of these genes raises question about the PRC2-independent activity of EZH2 but also about the regulation of PRC2/EZH2 activity at such promoters in the context of transient differentiating and proliferating cell states. PRC2-independent EZH2-mediated transcriptional activation was previously reported in prostate cancer and B-cell lymphoma through collaboration with transcriptional activators like E2F1 [71, 72]. It is plausible that PRC2/EZH2 activity at these transitionally expressed genes is inhibited by other factors as the PRC2 core subunit SUZ12 still colocalizes with EZH2 at some of these H3K27me3-free genes. In this context, an inhibitory role for the PCL subunits, PHF1 and MTF2 was demonstrated [73, 74]. MTF2 was shown to have both active and inhibitory roles in PRC2 function, however in a different cellular context. In ESCs, knockdown of *Mtf2* causes reduced H3K27me3 at specific PRC2 targets, like *Hox* genes and pluripotency TFs, yet global H3K27me3 levels increase compared to control [74, 75]. In contrast, in embryonic fibroblasts Mtf2 was shown activate the expression of *Cdkn2a* by suppressing the catalytic activity of PRC2 [74]. The knockdown of *Mtf2* showed increased H3K27me3 levels at the *Cdkn2a* locus, suggesting that Mtf2 can restrain PRC2 in certain contexts [74]. PHF1 was also shown to have PRC2 inhibitory function and one study showed that recognition of H3K36me3 by the PHF1 Tudor domain negatively regulates the enzymatic activity of PRC2 and lead to reduced levels of H3K27me3 *in vitro* and in HEK293T and K562 cells [73]. In a similar way in HSCs and prostate cancer cells, *PHF19* KD was associated with increased levels of H3K27me3, and with a repression of a subset PRC2 target genes suggesting that in some context PHF19 may have PRC2 inhibitory functions.

### PHF19 in germinal center and PC differentiation

Similar to EZH2 in the B-cell lineage, the expression of *PHF19* is low in mature naïve cells; reaches high levels in GC B-cells and then returns to low level in PCs and memory B-cells (Fig. 2C and D). Specifically, *PHF19* reaches its highest level of expression in centroblasts and pre-plasmablasts, two highly proliferative cell subsets (Fig. 2D). *PHF19* is also highly expressed by centrocytes and plasmablasts. *In-vitro*, *PHF19* is significantly upregulated after activation of memory B-cells by a combination of stimuli including CD40L, CpG oligodeoxynucleotide, IL2, IL10 and IL15 (Activated B-cells, act.BC) and by pre-plasmablast generated *in-vitro* (Fig. 2E). No study has comprehensively investigated the role of PHF19 (neither PHF1 nor MTF2) in B-cell development or plasma cell. However, using a bone marrow transplantation model of retrovirally transduced cells with *PHF19* or an empty vector, one study showed that PHF19 overexpression does not induce any difference in the percentages of mature B-cells or T-cells in the bone morrow or the spleen between mice overexpressing PHF19 and controls [76]. Mice overexpressing PHF19 in their blood-lineage cells showed, after sheep red blood cells (SRBC) immunization, increased percentages of GC B-cells and follicular helper T-cells (TFH) but also higher percentages of plasma cells in the spleen and the bone marrow [76]. Analysis of cell cycle and apoptosis of *ex-vivo* isolated GC B-cells and TFH cells suggested that PHF19 overexpression promoted their proliferation and survival potentially through the regulation of cell cycle genes, e.g. upregulation of *Ccnd2* [76]. However, the authors did not show how transduction of PHF19 impacted its expression levels in blood cells (in particular B-cells and T-cells) and the experimental model did not give insight into the impact of PHF19 on GC B-cell proliferation or plasma cell differentiation or whether it was intrinsic to these cells or was related to PHF19 overexpression in other blood cells. Connected with this, in rheumatoid arthritis, a chronic and systemic autoimmune disease affecting both joints and extra-articular tissues, and in which germinal center and autoantibodies play an important role, *PHF19* expression was shown to be higher in lymphocytes from synovial fluid and peripheral blood of rheumatoid arthritis patients compared to healthy controls [76]. Furthermore, genetic studies of rheumatoid arthritis heritability identified single nucleotide polymorphisms (SNPs) in the Chr 9q33.2 region involving *PHF19*, TRAF1 and C5 as genetic risk factors for rheumatoid arthritis [77, 78]. In particular, SNPs in the 3’ UTR region (rs1837) and in the third intron region (rs1056567), have been associated with increased risk of rheumatoid arthritis [78]. Overall, these data suggest that PHF19 play an important role in regulating GC and PC differentiation and relevant experimental model and comprehensive studies are needed to uncover this role.

## The role of PHF19 in Multiple Myeloma

### PRC2/EZH2 in Multiple Myeloma

PRC2 regulates expression programs important in cell differentiation, cell cycle and stem cell self-renewal and thus aberrant PRC2 activity might lead to cellular transformation. *EZH2*, is frequently overexpressed or mutated in a wide variety of cancer, including solid and hematological cancer [5, 6, 10]. In hematological malignancies, loss of function mutations of *EZH2* are reported in T-cell acute lymphocytic leukemia, myelodysplastic syndromes, and myeloproliferative neoplasms, however, gain of function mutations are observed in B-cell lymphomas [10]. In B-cell lymphomas the heterozygous point mutations affecting EZH2 at Y641 are in the catalytic domain. These mutations produce a neomorphic enzyme that is impaired in its ability to generate H3K27me1 but is more effective in generating H3K27me2/3 [62]. Mutated EZH2 in B-cell lymphoma enables persistent epigenetic silencing of genes involved in PC differentiation and negative regulation of cell cycle [62–64]. In MM, EZH2 is overexpressed compared to normal plasma cells and associated with adverse prognosis and high-risk clinical features but is generally not mutated [1, 79–83]. SUZ12 and EED are also overexpressed in MM cells, but are not associated with MM patient’s outcome [82, 83]. *EZH2* expression increases during the progression from MGUS to SMM to MM and reaching a maximum at the PCL stage and its expression correlates with the cell proliferation index [1, 70, 79, 80, 83, 84].

Several TFs involved in MM pathogenesis have been shown to regulate *EZH2* expression in lymphoid cells including E2F1, STAT3, c-MYC, and cREL [66, 85, 86]. IL-6 was shown to induce EZH2 protein expression in growth factor-dependent MM cell lines [87]. In gastric cancer, STAT3 was shown to bind to *EZH2* promotor and to increase EZH2 following IL6 stimulation [88]. E2F1 bound to the *EZH2* promoter and activated its expression in MM cells [89] and in GC B-cells [66]. The PI3K/Akt pathway was shown to modulate EZH2 and EZH1 levels via the AKT downstream effectors E2F1 and FOXO3, respectively. AKT inhibitors or bortezomib treatment were shown to downregulate EZH2 expression by abrogating E2F1 expression [86, 89]. EZH2 was also shown to function as a transcriptional activator [71, 90–93]. EZH2 enzymatic activity, stability, nuclear localization and assembly with the PRC2 components can be regulated by phosphorylation [93]. AKT was shown to phosphorylate EZH2 at serine 21 and suppress its methyltransferase activity by impeding EZH2 binding to histone H3, which results in a decrease of H3K27me3 and derepression of silenced genes [94]. In MM cells, EZH2 phosphorylation at serine 21 promotes cell growth and cell adhesion-mediated drug resistance [52, 95].

In MM, PRC2 target genes and the genome wide-distribution of H3K27me3 have been investigated in several studies. Downregulated genes in MGUS and MM compared to normal plasma cells have been shown to be enriched for PRC2/EZH2 target genes (A set of genes defined as PRC2/EZH2 targets in human embryonic fibroblasts) [96]. The study of H3K27me3 marks at five PRC2 targets commonly under-expressed in MM, namely, *CIITA*, *CXCL12*, *GATA2*, *CDH6* and *ICSBP/IRF8* showed an enrichment for this mark [96]. RNA-Seq combined with genome-wide profiling of H3K27me3 mark in MM cells has shown that H3K27me3-enriched genes overlap with PRC2/EZH2 target genes and with genes under-expressed in MM patients [12]. Specifically, H3K27me3-enriched genes in MM significantly overlap with under-expressed genes in ISS stage III by comparison with ISS stage I and II, were significantly enriched among under-expressed genes in plasma cell leukemia (PCL) and also significantly overlapped with genes under-expressed in MM patients with poor survival [12]. These data suggest that PRC2-mediated gene silencing is a mechanism of gene repression during MM progression. The number of bivalent genes defined by the presence of both H3K27me3 and H3K4me3 marks was increased in MM by comparison to normal plasma cells, with only a very small number of genes overlapping between the two entities. The bivalent genes had a similar expression profile to genes enriched for H3K27me3 marks alone and were enriched among the under-expressed genes in MM cell lines [12]. Notably, in germinal center B cells, EZH2 establish bivalency at genes involved in PC differentiation and negative regulation of the cell cycle promoting cell proliferation [62, 66]. Interestingly, upregulated genes after pharmacological inhibition of EZH2 showed overlap only with bivalent genes but not with H3K27me3-only enriched genes suggesting that bivalent genes might play an important role in MM pathogenesis and could be amenable to therapeutic targeting.

In MM cell lines, pharmacological inhibition of EZH2 decreased the global H3K27 methylation and had anti-myeloma effects both *in vitro* and *in vivo* [1, 12, 96–98]. Inhibition of EZH2 in MM leads to the downregulation of a c-MYC signature, the upregulation of cell cycle control genes including the CDK inhibitors *CDKN1A*/p21 and *CDKN2B*/p15 leading to cell cycle arrest and apoptosis [1, 98]. New evidence shows that in MM cell lines that the sensitivity to EZH2 inhibitor correlated with distinct metabolic signatures resulting from a dysregulation of genes involved in methionine cycling [99].

The t(4;14) subset of MM is notable for a global reduction of H3K27me3 levels. t(4;14) MM cells exhibit an increased expression of the H3K36 methyltransferase NSD2 resulting in increased levels of H3K36me2 and an imbalance in the distribution of the H3K36me2 and H3K27me3 marks [97]. H3K36me2 has been shown to inhibit both PRC2 binding to nucleosomes and in the methylation of histones [31, 100]. However, t(4;14) MM cells exhibit enhanced recruitment of EZH2 and enrichment of H3K27me3 at specific genomic loci involved in normal germinal center B-cells differentiation and in a subset of c-MYC targets genes in B-cells suggesting that EZH2-mediated repression of these genes may be important for NSD2-induced oncogenesis [97]. Accordingly, t(4;14)+ NSD2 overexpressing MM cell lines are more sensitive to EZH2 inhibition compared to cell lines with NSD2 low expression [97].

H3K27me3 marks are removed by the histone demethylases ubiquitously transcribed tetratricopeptide repeat X chromosome (UTX; also known as KDM6A) and jumonji domain-containing protein 3 (JMJD3; also known as KDM6B) [101]. In MM, *UTX/ KDM6A* is mutated or deleted in about 5% of primary MM cases [81]. In an isogenic cell line system, loss of UTX leads to a failure in the activation of the expression of some genes involved in cell growth, adhesion, survival and movement, and promotes proliferation, clonogenicity, adhesion, and tumorigenicity of MM cells *in-vitro* and *in-vivo* [102]. Interestingly, treatment of *UTX* null cells with EZH2 inhibitors reactivated the expression of nearly half of the genes repressed by UTX loss [102]. Furthermore, *UTX*-deficient MM cells showed increased sensitivity to EZH2 inhibition compared to MM cells expressing wild-type *UTX* [102]. These data from t(4;14)+ NSD2 overexpressing MM and from *UTX*-deficient MM suggest that EZH2 may have context-dependent oncogenic activities in MM.

### Transcriptional regulation of *PHF19* expression in Myeloma

In MM, the transcriptional regulation of *PHF19* gene is largely unexplored to date. While *PHF19* is located on chromosome 9q33.2, a chromosome frequently gained in MM, we (unpublished) and others [103] have shown only a negative correlation between trisomy Chr9 and *PHF19* expression indicating that high levels of *PHF19* are not induced by DNA copy number gain of Chr9.

**Fig. 3:**
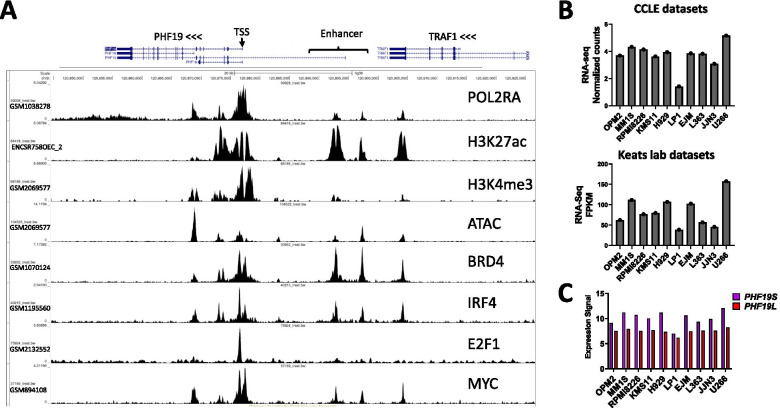
Chromatin marks and transcription factors at the *PHF19* locus and *PHF19* expression in human MM cell lines. **A** Snapshot from UCSC genome browser showing binding events within the *PHF19* locus in the MM1S cell line. **B** *PHF19* expression as reported using RNA-seq in the Cancer Cell Line Encyclopedia (CCLE) database [104] (https://depmap.org/portal/download/, accessed on 25 July 2021) (upper) and as reported in Keats lab dataset (https://www.keatslab.org/data-repository) (Lower). **C** PHF19 expression as reported using microarray in the CCLE database

In the MM1S cell line, our analysis of ChIP-seq data from the Cistrome databases showed that *PHF19* locus is decorated with active chromatin marks H3K27ac and H3K4me3 and POL2RA (Fig. 3A). ATAC-seq signals showed that the chromatin is accessible at the *PHF19* locus. Interestingly, BRD4, which bookmarks transcribed genes and active genes during mitosis is also identified at the PHF19 locus (Fig. 3A). The Mediator of RNA polymerase II transcription subunit 1 (MED1) is also present at the *PHF19* locus. ChIP-seq analysis of transcription factors show that the TSS of the *PHF19* gene is bound by E2F1, IRF4 and MYC (Fig. 3A). Further the *PHF19* gene was one of the 681 genes identified as associated with a cis-located super-enhancer region (coordinate on hg38 genome version chr9:120868122-120905040) [105] (Fig. 3A). This region is enriched for enhancer’s marks H3K27ac, H3K4me1 and accessible chromatin (ATAC-Seq) in the KMS11 MM cell line (not shown). Analysis of chromatin accessibility and TF binding at this Enhancer region showed the presence of IRF4, MYC and BRD4 at open chromatin spots (Fig. 3A). Notably, this enhancer was not identified in glioblastoma cell line U-87 MG or in small cell lung cancer cell line H2171 suggesting that it might be specific to plasma cells. Furthermore, this enhancer is different from the *trans*-located enhancer identified in Ewing sarcoma (See above, [47]). This epigenetic landscape in MM1S cell line, indicates that the *PHF19* locus is transcriptionally active and this correlates with the level of mRNA expression of *PHF19* (CCLE and Keats lab datasets) (Fig. 3B). Microarray RNA expression data of PHF19S and PHF19L from the CCLE datasets, shows that PHF19S is expressed at a higher level than PHF19L in MM cell lines (Fig. 3C). In our hands, this pattern of mRNA expression of PHF19S and PHF19L was also observed at the protein level by western blot in all the MM cell lines tested (Fig. 3C) (data not shown).

As IRF4 was one of the TF present at the enhancer and TSS of *PHF19*, we checked if *PHF19* expression is affected by the CRISPR mediated ablation of IRF4 in MM cell lines [106]. In this context, *PHF19* was not identified among the differentially expressed genes in MM1S, OPM2 or H929 cells suggesting that IRF4 is not directly involved in *PHF19* expression regulation. The analysis of published RNA-seq data from the use of the BRD4 inhibitor (AZD5153) vs control in MM1S cells identified *PHF19* as one of the downregulated genes [107]. The use of this BRD4 inhibitor also downregulated *PHF19* in other MM cell lines including IM9, MOLP8, OPM2 and RPMI8226 [107]. *PHF19* has been identified as a potential MYC target by ChIP-seq experiments [108], however, as far as we know there is no direct evidence that can link MYC to the regulation of *PHF19* expression (For example knockdown or pharmacologic inhibition of MYC followed by evaluation of *PHF19* expression levels). Both BRD4 inhibition [107] and IRF4-depletion lead to significant downregulation of MYC [106, 109]. However, BRD4 inhibition but not IRF4-depletion led to significant downregulation of *PHF19* expression. The pharmacological inhibition of H3K4me3 Lysine demethylase 5A (KDM5A), which also leads to a significant downregulation of MYC and MYC-targets, did not lead to significant changes in *PHF19* expression [110]. These data suggest that *PHF19* is not a target for MYC, however, the involvement of MYC in *PHF19* expression needs to be further resolved in MM.

### Clinical and biological consequences of *PHF19* overexpression in Multiple Myeloma

*PHF19* is overexpressed in MM and is higher in malignant PC compared to normal bone marrow PC [15, 52, 103]. The expression of *PHF19* increases with increasing disease stage and during the progression from the premalignant stages MGUS and smoldering MM into MM [15, 16, 52, 84, 103]. The highest *PHF19* expression is observed in the most aggressive variant, PCL. *PHF19* expression has been identified as the best individual predictor of risk in a MM DREAM challenge [15]. We note, however, that *PHF19* was not identified among the prognostic gene expression profiles of MM including the GEP70 [111], EMC92 [112] and the IFM15 [113]. In a recent study aiming at establishing a transcriptional regulatory network in order to predict disease progression, PHF19 was identified as a member of a genetic program (Program 68) that predicts high-risk behavior and early relapse [114]. Along with other genes in this program (e.g., *CKS1B*, *PCNA*, *E2F1*, *FOXM1*), *PHF19* expression was associated with cell proliferation and was among the best genes predictors of high-risk status [114]. In a recent preprint study analyzing single cell RNA-seq of FACS-sorted CD138+ MM cells using Seurat, showed that MM cells cluster into five major subtypes (clusters) with the cell subtype-2 expressing high level of *PHF19* compared to the others subtypes [115]. This observation suggests that only a subpopulation of MM tumor cells express high levels of *PHF19* bringing the association between PHF19 and MM aggressiveness to a cellular level. The presence of the *PHF19*high myeloma cell subtype-2 is showed to be associated with myeloma progression using a new deep learning tools called DEGAS [115]. Interestingly, this *PHF19* high myeloma cell subtype also exhibited high expression of genes involved in cell cycle including *HELLS*, *EZH2*, *TYMS*, *ZWINT*, and *MKI67* [115].

Multiple lines of evidence suggest PHF19 is a mediator of adverse prognosis because of its potential to control the expression of genes associated with drug resistance and proliferation [15, 22, 30, 35, 36, 52, 84, 103]. Our team identified PHF19 and EZH2 among the deregulated genes between SMM and MM in the seven major subtypes of MM (D1-HRD, D2, CCND1-11q13, CCND3-6p21, MMSET, MAF and MAFB) [84]. In a global challenge to identify biomarkers of high-risk behavior in MM, bioinformatic teams from around the world competed to identify expression patterns that correlated with poor outcome from eight data sets totaling 2447 patients [15]. This effort identified *PHF19* as the most significant contributor, more so even than *NSD2* (*MMSET*), *CKS1B* and *MAF* expression, known mediators of MM risk [15]. Incorporation of *PHF19* and *MMSET* expression with age and ISS identified a simple model of high-risk MM [15] and *PHF19* high expression is associated with poor prognosis [15, 16, 52, 103]. Higher *PHF19* expression is significantly associated with multiple high-risk genetic factors such as IGH translocation groups, non-hyperdiploidy, *TP53* mutations and the overall number of drivers per sample [15]. Furthermore, well known proliferation genes including *EZH2*, *MCM4*, *TYMS*, *AURKB*, *CHEK1*, *MCM2*, *ZWINT*, *CCNA2* and *BIRC5* were highly correlated with *PHF19* expression [15].

Using an shRNA loss-of-function approach, several study have shown that MM cell lines with different genetic background (ARP1, OCI-My5, JJN3, MM1S, L363, RPMI8226, H929 and KMS11) are dependent on PHF19 for their proliferation [15, 16, 52, 103]; however, the mechanism by which this is mediated remains undefined. The knockdown of both PHF19L and PHF19S or only PHF19L has been shown to diminish the cell growth and colony formation of several MM cell lines [16, 52, 103], however, only the ectopic rescue of PHF19L restored normal cell growth and colony formation [103]. Mice xenografted with MM cell line knocked down for PHF19L showed suppressed tumor growth and prolonged survival compared with controls [16, 52, 103]. In the study by Yu T et al. [52], the ectopic overexpression of PHF19L in ARP1 and OCI-My5 MM cells, increased cell growth and induced drug resistance to bortezomib, epirubicin, or melphalan and promoted tumor growth in vivo [52]. Cell cycle analysis showed that knockdown of PHF19 lead to cell cycle defect characterized by the accumulation of the cell in G0/G1 of the cell cycle [15, 103].

**Fig. 4:**
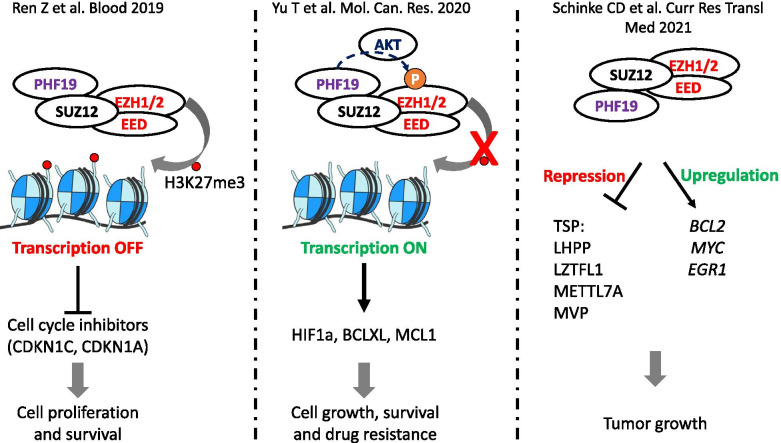
Schema showing suggested mechanism of action of PHF19 overexpression in MM cell lines. Ren Z et al. showed that PHF19 promotes PRC2 activity and represses cell cycle inhibitor genes. Yu T et al. showed that PHF19 impedes PRC2 activity by promoting the phosphorylation of EZH2 via AKT pathway leading to EZH2 inactivation leading to an increase in expression of genes that play an important role in MM. Schinke C et al. showed that PHF19 repressed the expression of tumor suppressor protein (TSP) and upregulated the expression of pro-survival and proliferation genes

Importantly, mechanistic investigation of PHF19 function in MM has given conflicting results, (Fig. 4). On one hand, Ren et al. [103] showed that PHF19 is critical for the maintenance of the H3K27me3 landscape across the chromatin, with the exception of CpG rich promoters and that it represses cell cycle inhibitor genes e.g. *CDKN1A/C* and JAK-STAT pathway genes. In contrast, Yu et al. [52] reported that *PHF19* depletion promotes the phosphorylation of EZH2 leading to EZH2 inactivation via the PI3K/AKT pathway, thus causing a decrease in H3K27me3 and H3K27me2 marks thus promoting the expression of genes involved in MM cell survival, proliferation and conferring drug resistance via JUN, KLF, RELB, HIF1α, BCLXL and MCL1. Schinke et al. showed that *PHF19* KD leads to the downregulation of major cancer players such as BCL2, MYC and EGR1 in ARP1 and JJN3 cell lines and to the downregulation of genes within the JAK/STAT pathway in JJN3 cell line only suggesting that PHF19 is somehow involved in the upregulation of these genes [16]. Despite these differences, the three studies show that *PHF19* overexpression is associated with more aggressive proliferation and drug resistance in MM cell lines making it critical to resolve the key mechanism by which this is mediated.

In MM, inactivation of EZH2 by phosphorylation was shown to lead to cell-adhesion mediated drug resistance to doxorubicin and the alkylating agent 4-OHCY [95]. Adhesion and cell-cell contact signals activated the PI3K/Akt pathway to induce the phosphorylation of EZH2 on the Ser-21, leading to H3K27 hypomethylation resulting in the activation of antiapoptotic genes including IGF1 [95]. Pharmacological and genetic inhibition of the IGF1R-PI3K-AKT pathway reverses CAM-DR by promoting EZH2 dephosphorylation and H3K27 hypermethylation both *in vitro* and in refractory murine MM models [95]. EZH2 is also phosphorylated by CDK1 on Thr-345 and Thr-487 that promotes EZH2 ubiquitination and subsequent degradation by the proteasome [116] or disrupted EZH2 binding with the other PRC2 components SUZ12 and EED, and thereby inhibited EZH2 methyltransferase activity [117]. AKT pathway is one of the upstream regulators of *PHF19* expression [34, 43] and PHF19 promotes AKT pathway signaling [45] and CDK1 seems to be one of the downstream target of PHF19 activity in MM (See below). These observations suggest that PHF19 might regulate PRC2/EZH2 activity via an effect on mediating the expression of CDK1 and the promotion of AKT activity.

### PHF19 is involved in the regulation of genes important in cell cycle and the genetic stability of MM cells

**Fig. 5:**
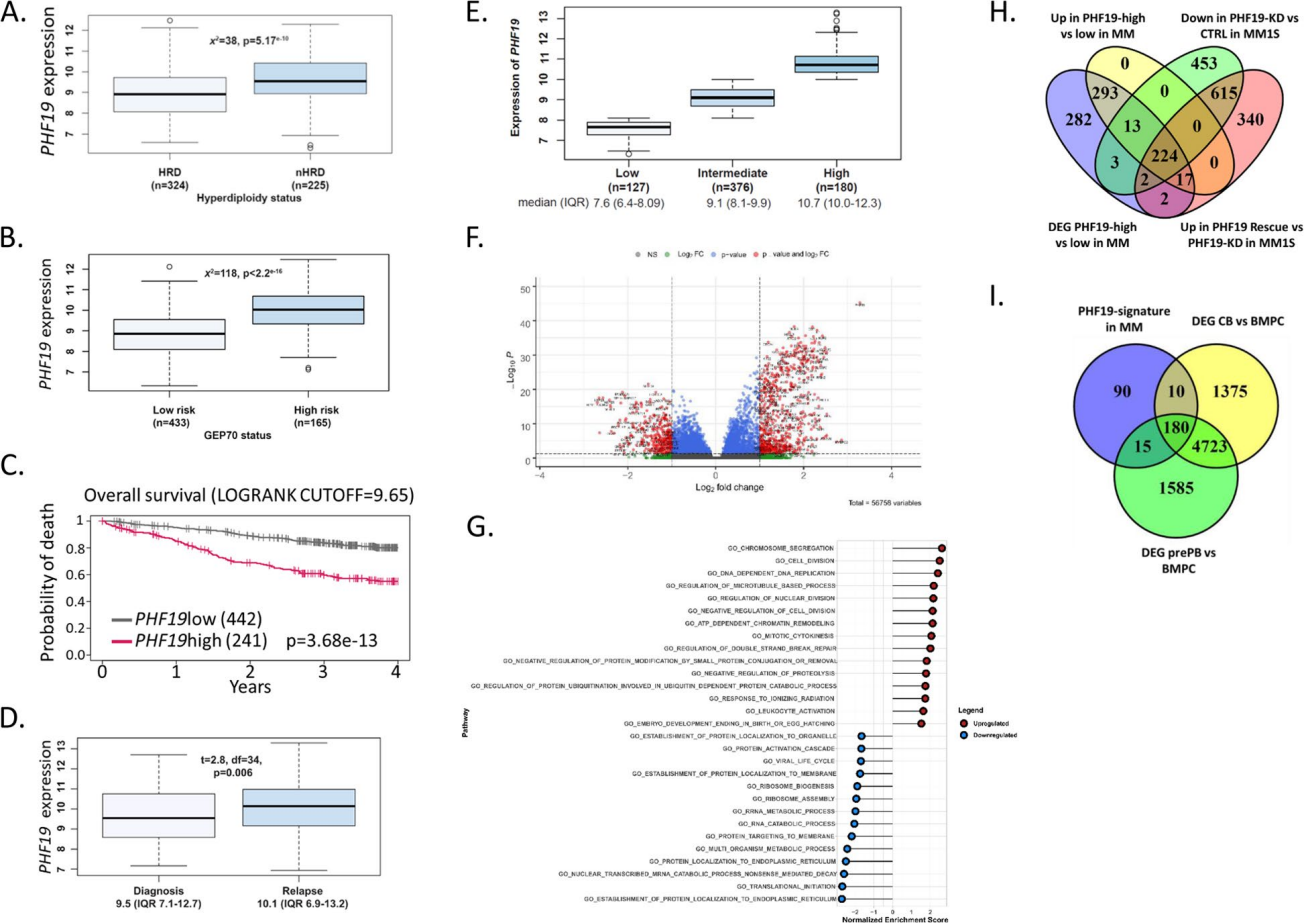
High *PHF19* level is associated with high-risk and PHF19 transcriptional signature in MM suggests that it might regulate cell cycle progression. **A and B** *PHF19* is expressed in all MM subtypes with higher expression levels seen in the non-hyperdiploid subgroups (X2=38, *p*=5.7e^-10^) and HR patients defined by GEP70. Interestingly, *PHF19* expression is significantly higher at relapse (t=2.8, df=34, *p*=0.006). **C** Using a logrank test we identify a PHF19 expression level of 9.65 as an optimal cut point for overall survival (OS) splitting the population into high and low *PHF19* expressing groups. The OS of patients with elevated *PHF19* expression is significantly shorter than patients with lower PHF19 expression (HR=2.98 (2.2-4), *p*=3.68e^-13^. **D** *PHF19* expression is significantly higher at relapse (t=2.8, df=34, *p*=0.006). **E** *PHF19*high and low groups were defined using an elbow test. **F** Volcano plot showing genes differentially expressed between *PHF19*high and *PHF19*low MM samples. Analysis identified 835 differentially expressed genes (DEG) (Fold change >2, FDR < 0.05), with 547 (65%) upregulated and 288 (35%) downregulated genes. **G** Gene set enrichment analysis (GSEA) analysis of the differentially expressed genes between *PHF19* high and low MM samples (Gene ontology (GO)). **H** Venn diagram showing the overlap of: 1- DEG between *PHF19*high and *PHF19*low MM samples, 2- Downregulated genes between *PHF19*-KD and *PHF19*-WT in MM1S cell line (i.e. genes upregulated by PHF19), 3- Upregulated genes between PHF19-overexpression (rescue) and *PHF19*-KD in MM1S cell line (i.e. genes upregulated by PHF19) and 4- upregulated genes between *PHF19*high and *PHF19*low MM samples. **I** Venn diagram showing the overlap of: 1- the 294 genes defining PHF19 signature in MM, 2- DEG between centroblasts (CB) and bone marrow plasma cells (BMPC) and 3- DEG between preplasmablasts (PrePB) and BMPC

In order to define the transcriptional program and biological pathways promoted by PHF19, we analyzed the gene expression data from the Multiple Myeloma Research Foundation (MMRF) CoMMpass study of 683 newly diagnosed MM and 35 relapse patients, (Fig. 5). *PHF19* is expressed in all MM subtypes with higher expression levels seen in the non-hyperdiploid subgroups (*χ*^*2*^=38, p=5.7.10^-10^) and HR patients defined by GEP70 (Fig. 5A and B). Using the logrank test we identified a PHF19 expression level (of 9.65) as an optimal cut point for overall survival (OS) splitting the population into high and low *PHF19* expressing groups. The cases with elevated *PHF19* expression (35%, 241/683) were associated with inferior OS (Fig. 5C). *PHF19* expression was significantly higher at relapse (Fig. 5D). These data confirm and extend the results of previous reports using different MM series [15, 16, 52, 103, 118].

*PHF19* high and low groups were also defined using an elbow test (Fig. 5E). Using this cutpoint to define the transcriptional signature associated with high *PHF19* expression we compared the gene expression profile of high vs low *PHF19* samples and identified 835 differentially expressed genes (DEG) (Fold change >2, FDR < 0.05), with 547 (65%) upregulated and 288 (35%) downregulated genes (Fig. 5F). GSEA showed that these genes are enriched for cell division, DNA replication and chromosome segregation processes (Fig. 5G). To directly link the regulation of expression of these genes/pathways to PHF19, we analyzed RNA-seq data from the MM1S cell line in which *PHF19* was knocked down by shRNA and identified differentially expressed genes between *PHF19*-KD cells and *PHF19*-WT [103]. Overlapping the DEG from this analysis in MM1S cell lines and from patients’ samples, we identified 294 common genes and defined them as the PHF19 transcriptional signature of MM (Supplementary Table 2). Interestingly, 224 of these genes (82%) were upregulated in *PHF19*-high vs low MM samples, are downregulated after PHF19 depletion in the MM1S cells and are upregulated after restoring the expression of PHF19 in depleted cells (Fig. 5H). These data suggest that PHF19 is involved in the transcriptional activation of these genes.

We analyzed the RNA-seq data of HSCs from a *PHF19* knockout mouse model [56]. PHF19 in HSCs was shown to control the balance between quiescence and proliferation as well as the balance between self-renewal, differentiation and maintenance [56]. In total 52 out of the 294 genes identified in our MM analysis were also found to be DE between *Phf19*-WT and *Phf19*-KO mouse HSCs. Interestingly, 43 out of the 52 genes, were downregulated after *PHF19*-depletion in MM cells and in mouse HSCs suggesting that PHF19 positively regulate the transcription of these genes. These 43 genes are mostly involved in cell cycle progression and include genes like *TYMS*, *AURKB*, *CCNA2* and *BIRC5* (Supplementary Table 3) that significantly correlated with high PHF19 expression in MM [15]. This observation suggests that PHF19 activity may stimulate the expression of cell cycle associated genes in different cellular context including malignant PCs affecting their proliferation and maintenance and leading to therapeutic resistance and HR behavior.

In normal physiology, PHF19 may be involved in regulating late-stage B-cell development so its aberrant expression could be relevant to the pathology of MM. To address this idea we explored the relevance of the 294 genes in GC B-cell and plasma cells (PC) differentiation program by analyzing their expression in normal human GC B-cells (centroblasts) and PCs. *PHF19* expression was the lowest in quiescent mature naïve B-cell and PC and the highest in proliferating centroblast and pre-plasmablast (Fig. 2D). We show that 175 genes and 180 genes (about 61%) of these 294 genes are shared with DEG between centroblasts or pre-plasmablast and mature bone marrow PC, respectively (Fig. 5L). The majority of these genes are involved in cell cycle regulation.

**Fig. 6:**
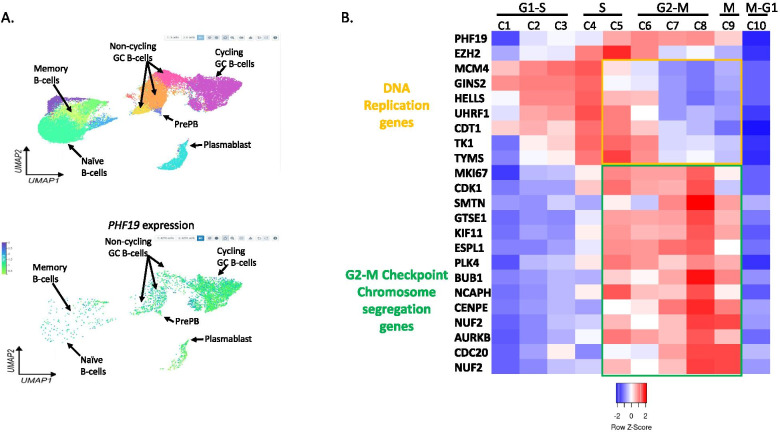
Single cell RNA-seq data from germinal centers links *PHF19* expression modulation to cell cycle. **A** *PHF19* expression is the highest in cycling GC B-cells and preplasmablasts and plasmablasts. Upper panel shows a UMAP projection and cluster identification from single cell RNAseq profiles of human B cells maturation in tonsils [120]. Lower panel shows UMAP plot showing expression of *PHF19* in the different B cells clusters as shown in the upper panel (www.tonsilimmune.org/, accessed on 16 June 2021). **B** Heatmap displaying the relative expression fold change (log2) of *PHF19* and selected genes in clusters of dark zone B cells representing different stages of the cell cycle (data are from [119]. Ten clusters were identified by PhenoGraph in dark zone GC B cells based on the expression of genes associated with the S-G2-M stages of the cell cycle: three clusters of cells transitioning from G1 to S phase (C1, C2 and C3), two clusters in the S-phase (C4 and C5), three clusters of cells transitioning from G2 to M phase (C6, C7 and C8), one cluster in the M-phase (C9) and one cluster of cells transitioning from M to G1 phase (C10). The differential expression analysis is performed by comparing each cluster to all the others. *PHF19* expression peaks in the G2-M phase of the cell cycle and this is followed by the modulation of several genes of the PHF19 transcriptional signature

Analysis of published single cell RNA-seq data [119, 120] showed that *PHF19* is upregulated by cycling GC B-cells and plasmablasts compared to non-cycling GC, memory B-cells and naïve B-cells (Fig. 6A). Specifically, in GC B-cells the expression of *PHF19* peaks in the G2-M phase of the cell cycle and this is followed by the modulation of several genes of the PHF19 transcriptional signature that we identified in MM and that are involved in cell cycle regulation including *PLK4*, *BUB1*, *CENPE*, *NUF2* and *AURKB* (Fig. 6B). This pattern of expression suggests that PHF19 could play a role in the transcriptional regulation of genes specific to the G2-M checkpoint, the mitotic cell cycle transition by which a cell in G2 commits to M phase and where the cell must maintain the integrity and the proper segregation of the recently duplicated chromosomes. The deregulation of the expression of these genes by PHF19 overexpression may lead to abnormal cell cycle, increased cellular proliferation and genomic instability. The G2M checkpoint plays a critical role in cell cycle and its deregulation can lead to cancer. Cancers with high activity of G2M pathway genes tend to be more aggressive. These data suggest that PHF19 overexpression in PC may re-induce the expression of genes involved in proliferation and cell cycle, a situation that would be biologically relevant to the malignant behavior of a cell type otherwise destined for quiescence or apoptosis when the initial immune response is no longer required. Furthermore, single cell RNA-seq analysis identified that only a subpopulation of MM malignant cells overexpress *PHF19* [115]. Interestingly, this *PHF19*high myeloma cell subpopulation seems to be the cell cycling fraction as they also exhibited high expression of genes involved in cell cycling including *HELLS*, *EZH2*, *TYMS*, *ZWINT*, and *MKI67* [115].

**Fig. 7:**
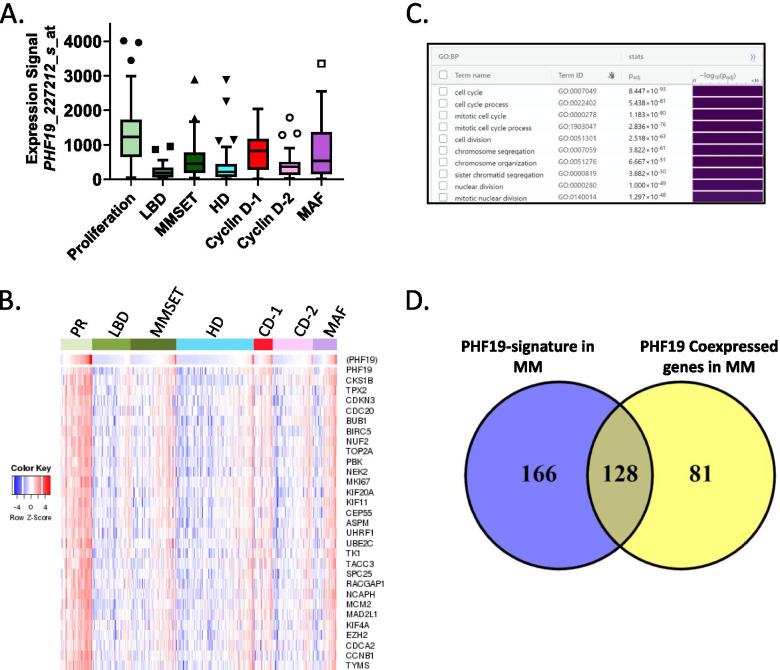
*PHF19* expression is associated with cell proliferation in MM. **A** PHF19 expression in molecular subtype of MM, proliferation subgroup (PR, *n*=47), low bone disease (LBD, *n*=58), MMSET (*n*=68), hyperdiploidy (HD, *n*=116), Cyclin D-1 (CD-1, *n*=28), Cyclin D-2 (CD-2, *n*=60) and MAF (*n*=37). **B** Coexpression analysis of PHF19 using GenomicScape database [55] (http://genomicscape.com/microarray/expression.php, accessed on 03 September 2021). Heat maps showing the top 30 genes positively correlated with PHF19 in a UAMS cohort [121]. **C** Gene set enrichment analysis (GSEA) analysis was performed using g:Profiler web tool (Biological Process ontology, GO:BP, https://biit.cs.ut.ee/gprofiler/gost). **D** Venn diagram showing the overlap between the 294 genes identified as the PHF19 transcriptional signature of MM and the 209 genes that positively correlate with expression of *PHF19*

Molecular classification of MM by unsupervised hierarchic clustering of mRNA expression profiles in CD138-enriched plasma cells from 414 newly diagnosed patients identified seven disease subtypes influenced by known genetic lesions [121]. These seven subgroups are MAF (c-MAF and MAFB), Cyclin D-1 (CCND1), Cyclin D-2 (CCND1 and CCND3), MMSET-activating translocations, hyperdiploidy (HD), low bone disease and proliferation subgroup (PR). *PHF19* expression is the highest in the PR subgroup (Fig. 7A). Using Genomicscape [55] platform we performed coexpression analysis and found that 209 genes positively correlated with *PHF19* expression (Pearson correlation coefficient ≥ 0.5 and *p* value ≤ 0.05). The heatmap shows the top 30 coexpressed genes with *PHF19* in the seven molecular subgroups (Fig. 7B). GSEA analysis (Biological Process ontology, GO:BP) of these 209 genes showed an enrichment for cell cycle regulation, the integrity of mitosis and chromosome segregation processes (Fig. 7C). Overlapping the genes from this analysis and the 294 genes identified as the PHF19 transcriptional signature of MM (see above) showed that 128 of the 209 genes (61%) are common (Fig. 7D).

Overall, these data strongly implicate PHF19 in the regulation of genes important in the proliferation, genetic stability and cell cycle of cells making it highly relevant to the basis of HR behavior. Thus, if we are to understand how HR behavior is mediated it is essential to understand the cellular and epigenetic mechanisms by which PHF19 promotes aggressive disease behavior; understanding these mechanisms will allow us to design of therapeutic strategies able to target aggressive disease.

## Conclusions and perspectives

Overall, data from different cell-context and experimental design strongly implicate PHF19 in the regulation of genes important in cell cycle regulation and the genetic stability of cancer cells making it highly relevant to the basis of HR MM behavior. In MM, the control mechanism for the abnormal H3K27me3 patterns is elusive and the role played by PHF19 as a regulator of PRC2 activity in this process is still not clear. Until recently the PRC complex had been described as an epigenetic repressor, however, emerging evidence suggests a scenario in which PRC proteins can also have a dynamic effect in mediating gene expression levels. Thus, while PHF19 is known to recruit and increase the transcriptional repressive activity of PRC2 through an increased deposition of the repressive mark H3K27me3, new studies in MM, prostate cancer and mouse hematopoietic stem cells have shown that PHF19-depletion is associated with a focal increase of H3K27me3 mark and that PHF19 may also play a role in the activation of gene expression. Experimentally the conflicting data highlight the limitations of MM cell lines for the assessment of the mode of action of epigenetically active genes and suggest the need to investigate PHF19 mechanisms in a more appropriate biological model. The current data indicate that PHF19 either plays a role in context-dependent regulation of PRC2 activity or alternatively that it may have activity independent of PRC2. To differentiate these two possibilities, it is crucial to identify the genes that are regulated by PHF19 and then to investigate how these genes are epigenetically controlled in a relevant model system i.e germinal center B-cells, plasmablasts or plasma cells. Furthermore, the role of PHF19 short isoform still to be clarified in MM as this form contains both the Tudor domain and the first PHD domain and is present in the nucleus of cells meaning it still can interact with H3K36me3 and have an impact on the long isoform function. The roles and molecular mechanism that may be established by both PHF19 isoform should be more clearly investigated.

## Supplementary Information

**Additional file 1: Supplementary Table 1.** Transcripts and protein isoforms of *PHF19* reported in Ensembl, NCBI and protein human atlas databases.

**Additional file 2: Supplementary Table 2.** Genes that represent PHF19 transcriptional signature in multiple myeloma.

**Additional file 3: Supplementary Table 3.** Genes potentially regulated by PHF19 in hematopoietic stem cells and in MM cells.

## Data Availability

The material supporting the conclusion of this review has been included within the article. The datasets analyzed during the current study are available in the following repository: http://dbtoolkit.cistrome.org/ http://servers.binf.ku.dk/bloodspot/ http://genomicscape.com/microarray/expression.php https://depmap.org/portal/download/ https://www.keatslab.org/data-repository www.tonsilimmune.org/

## Abbreviations

act.BC: Activated B-cells
DEG: Differentially expressed genes
EED: Embryonic ectoderm development
EZH: Enhancer of Zeste Homolog
GC: Germinal center
GSEA: Gene Set enrichment analysis
HD: Hyperdiploidy
H3K27me3: Tri-methylation of the lysine (K) 27 on histone 3 H3
HR: High-risk
HSC: Hematopoitic stem cell
KD: Knockdown
mESCs: Mouse embryonic stem cells
MGUS: Monoclonal gammopathy of undetermined significance
MM: Multiple myeloma
MMRF: Multiple Myeloma Research Foundation
mRNA: Messenger ribonucleic acid
PC: Plasma cells
PCL: Plasma cell leukemia
PHF: Plant Homeodomain Finger Protein
PRC2: Polycomb Repressive Complex 2
SMM: Smoldering multiple myeloma
SRBC: Sheep red blood cells
SUZ12: Suppressor of zeste 12
TFs: Transcription factors
