# Supplementary material for: Insights into high-risk multiple myeloma from an analysis of the role of PHF19 in cancer
Source: J Exp Clin Cancer Res. 2021 Dec 2;40:380. doi: 10.1186/s13046-021-02185-1 (PMC8638425; doi:10.1186/s13046-021-02185-1)
Supplement: Supplementary file 1 — Additional file 1: Supplementary Table 1. Transcripts and protein isoforms of PHF19 reported in Ensembl, NCBI and protein human atlas databases. [file 13046_2021_2185_MOESM1_ESM.docx]

Supplementary table 1: transcripts and protein isoforms of *PHF19* reported in Ensembl, NCBI and protein human atlas databases.

| TRANSCRIPT_ID  Ensembl | TRANSCRIPT_ID  RefSeq NCBI | Splice_Variant | UniProt_ID | Amino Acid | Molecular Weight |
| --- | --- | --- | --- | --- | --- |
| ENST00000373896 | NM_015651.3 | PHF19-202  Long Isoform | Q5T6S3 | 580 | 70 KDa |
| ENST00000312189 | NM_001009936.3 | PHF19-201  Short isoform | Q5T6S3 | 207 | 22.5 KDa |
| ENST00000616568 | NM_001286840.1 | PHF19-214 | A0A087X169 | 599 | 72 KDa |
| ENST00000419155 | NM_001286842.1 | PHF19-203 | F5H8K3 | 371 | 42.2 KDa |
| ENST00000456291 | NM_001286843.2 | PHF19-207 | X6RER8 | 106 | 11.9 KDa |
| ENST00000453868 |  | PHF19-206 | B0QZ50 | 173 | 20.2 KDa |
| ENST00000436309 |  | PHF19-204 | B0QZ72 | 165 | 18.2 KDa |
| ENST00000439674 |  | PHF19-205 | B0QZ51 | 60 | 7.4 KDa |
