# Supplementary material for: Insights into high-risk multiple myeloma from an analysis of the role of PHF19 in cancer
Source: J Exp Clin Cancer Res. 2021 Dec 2;40:380. doi: 10.1186/s13046-021-02185-1 (PMC8638425; doi:10.1186/s13046-021-02185-1)
Supplement: Supplementary file 2 — Additional file 2: Supplementary Table 2. Genes that represent PHF19 transcriptional signature in multiple myeloma. [file 13046_2021_2185_MOESM2_ESM.docx]

Supplementary table 2: Genes that represent PHF19 transcriptional signature in multiple myeloma.

| PHF19 | KIF2C | WDHD1 | EZH2 | CH507-145C22.1 | SUSD1 |
| --- | --- | --- | --- | --- | --- |
| MYBPC2 | CDCA8 | GGH | CPNE4 | IGFBP6 | EFHC2 |
| PLD4 | CCNA2 | NCAPG | ESCO2 | HBD | TIMELESS |
| WEE1 | FLNA | HMGB3 | CENPU | CCDC34 | AC069277.2 |
| IQGAP3 | HSD11B1 | ADAM23 | LPGAT1 | AC004381.6 | MCM5 |
| MKI67 | CCNB2 | SKA3 | TNRC6C-AS1 | HMMR | PRC1 |
| KIF4A | USP13 | FAM131C | DIAPH3 | ATAD5 | IRX5 |
| NUF2 | EXO1 | FRMPD3 | CCDC15 | ARNTL2 | LAMB3 |
| TOP2A | CDC45 | HELLS | PTTG1 | CDCA4 | XRCC2 |
| RRM2 | PKMYT1 | KCNK13 | FAM72A | CENPH | CASC5 |
| DLGAP5 | CKAP2L | CENPE | MYBL2 | RP11-443B20.1 | DEPDC1B |
| DTL | NUSAP1 | TCF19 | ARHGAP11A | GSG2 | GINS3 |
| TPX2 | KIF14 | KIF15 | GINS4 | LMNB1 | CTD-2314B22.1 |
| CH507-24F1.1 | GAS1 | GINS1 | ASF1B | KIAA0101 | RP11-85I17.2 |
| CENPF | CENPA | SPAG5 | CALHM3 | CHAF1A | PSMC3IP |
| TK1 | APOBEC3B | AP000251.3 | GRB14 | FANCA | BRCA1 |
| E2F8 | NCAPH | CDKN3 | CKS1B | DPYSL2 | ARHGAP11B |
| CEP55 | MCM2 | CLSPN | STEAP1 | ACOT7 | PARPBP |
| SHCBP1 | CDCA2 | FAM72B | IGFBP7 | CHTF18 | CHEK1 |
| TYMS | ANLN | FAM83D | CBLN2 | RIBC2 | CLU |
| UBE2C | AC108463.2 | SPC25 | SGOL1 | NRGN | MCIDAS |
| CDC20 | LEFTY2 | TLR7 | KIF18A | KIF22 | AMIGO2 |
| ASPM | BUB1 | C16orf59 | RAD51 | ZMIZ1-AS1 | PARK2 |
| E2F1 | MCM10 | ZNF367 | OIP5 | MCM6 | BIN1 |
| E2F2 | CDCA5 | POLE2 | FAM64A | WDR76 | HIST1H2AC |
| CDK1 | PLK4 | FOXO6 | SYT1 | PCNA | PNPLA7 |
| CDT1 | ESPL1 | CCNB1 | C2orf48 | STIL | LY86 |
| ZWINT | KIF11 | CENPI | POLQ | KIF20B | ZNF69 |
| KIF18B | CDCA7 | LINC01105 | NCAPG2 | CHAF1B | GRIK4 |
| STMN1 | FOXM1 | MND1 | CDC25A | CCNF | CCNE1 |
| PBK | IER5L | STK39 | CENPW | DDX60 | TENM4 |
| NEK2 | PAQR4 | FZD8 | CDC6 | DRP2 | TMEM184A |
| TMSB15A | SKA1 | RECQL4 | CENPK | SLC44A2 | PECAM1 |
| BIRC5 | KIF23 | TMPO-AS1 | AC108463.1 | DCK | IGSF11 |
| CH507-154B10.2 | PLK1 | CBX2 | RHBDL3 | AUNIP | ZDHHC11B |
| LY6E | CIT | RAB37 | RAD51AP1 | C1orf112 | ENPP5 |
| RMI2 | GTSE1 | FAM111B | TRAIP | KCNN2 | LRRN2 |
| DEK | MCM4 | MYLK2 | POC1A | MSH2 | ECHDC2 |
| DSCC1 | MELK | WDR62 | EME1 | MCM3 | MYOF |
| E2F7 | TICRR | ASB2 | DNMT3B | WHSC1 | BIRC3 |
| CH507-145C22.3 | UBE2T | PRR11 | CDC7 | WDR34 | AF131217.1 |
| KIF20A | NEIL3 | ORC1 | TACC3 | KIF24 | TMEM156 |
| KIFC1 | UHRF1 | C17orf53 | CDC25C | CCDC150 | P3H3 |
| HJURP | CFH | SPC24 | SGOL2 | C21orf58 | GPR176 |
| BUB1B | CTD-2510F5.4 | IL24 | FANCI | XXbac-BPG252P9.9 | TMEM63C |
| AURKB | NDC80 | CENPM | LAIR1 | BRI3BP | RP11-800A3.4 |
| TROAP | TTK | AURKA | BRIP1 | KIFC3 | FAM101B |
| GINS2 | ATAD2 | DMC1 | RACGAP1 | RAB15 | SCUBE1 |
| DEPDC1 | RAD54L | TRIP13 | MAD2L1 | TMEM158 | CCRL2 |
