# Supplementary material for: Insights into high-risk multiple myeloma from an analysis of the role of PHF19 in cancer
Source: J Exp Clin Cancer Res. 2021 Dec 2;40:380. doi: 10.1186/s13046-021-02185-1 (PMC8638425; doi:10.1186/s13046-021-02185-1)
Supplement: Supplementary file 3 — Additional file 3: Supplementary Table 3. Genes potentially regulated by PHF19 in hematopoietic stem cells and in MM cells. [file 13046_2021_2185_MOESM3_ESM.docx]

Supplementary table 3: Genes potentially regulated by PHF19 in hematopoietic stem cells and in MM cells.

| ASF1B | **CENPA** | **KIF20B** | SHCBP1 |
| --- | --- | --- | --- |
| **ASPM** | **CENPE** | LMNB1 | SKA1 |
| AUNIP | **CENPW** | **MAD2L1** | SPC24 |
| **AURKB** | CKAP2L | **MKI67** | STIL |
| BIN1 | **CKS1B** | MND1 | **TYMS** |
| **BIRC5** | CLSPN | MYBL2 | **UBE2C** |
| BRIP1 | DLGAP5 | **NUF2** | WDR76 |
| **CCNA2** | **E2F8** | ORC1 |  |
| **CCNB1** | ESCO2 | PARPBP |  |
| **CDC25C** | FAM72A | **POLE2** |  |
| **CDK1** | **GINS1** | PSMC3IP |  |
| **SGOL1** | **KIF14** | RRM2 |  |
